# Deciphering pleiotropy: How complex genes regulate behavior

**DOI:** 10.1080/19420889.2018.1447743

**Published:** 2018-03-23

**Authors:** Ina Anreiter, Marla B. Sokolowski

**Affiliations:** a Department of Ecology and Evolutionary Biology, University of Toronto, Toronto, Ontario, Canada; b Child and Brain Development Program, Canadian Institute for Advanced Research (CIFAR), MaRS Centre, Toronto, Ontario, Canada

**Keywords:** Behavior, pleiotropy, *foraging*, gene regulation

## Abstract

The genetic underpinnings of animal behavior are exceedingly complex. Behavioral phenotypes are commonly regulated by many genes, and the behavioral effects of a gene often dependent on environmental conditions and genetic background. To complicate the study of behavioral genetics further, many genes that regulate behavioral phenotypes are themselves very complex genes, with several gene products and functions. One example of such a complex gene is the *foraging* gene in *D. melanogaster*. *foraging* influences many behaviors in the fruit fly, and the key to its effects likely lies in its complex molecular structure. We've recently found that expression levels of a small subset of transcripts of the *foraging* gene underlie the behavioral differences seen in adult foraging patterns of the rover and sitter *D. melanogaster* strains. Here we comment on the larger implications of this and other findings on gene regulation and pleiotropy in behavior.

The fruit fly *foraging* gene encodes a cGMP-dependent protein kinase that is involved in regulating many phenotypes, including feeding behavior [1–3], metabolic phenotypes (e.g. fat storage and starvation resistance) [3], learning and memory [4,5], and sleep [6] (Fig. 1). Interestingly, when it comes to ‘how’ *foraging* affects these different phenotypes, there is no simple answer. *foraging* has four promoters, twenty-one transcripts, and nine distinct protein isoforms, all of which share a common 3′ protein kinase domain but differ in their substrate binding and regulatory domains [3]. This complex molecular structure allows for the production of different gene products at different times in different tissues, likely through the use of different transcriptional regulators. A spatially and temporally restricted expression pattern of different gene products is one way in which one gene can regulate several independent phenotypes (pleiotropy).

**Figure 1.**
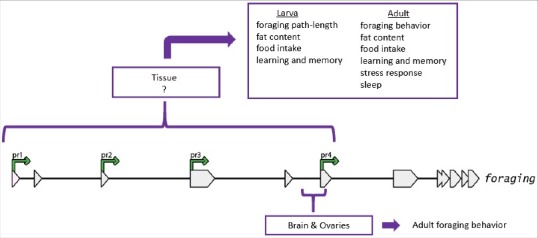
Pleiotropic effects of *foraging.* *foraging* regulates several independent larval and adult phenotypes in *D. melanogaster*. Variation in adult foraging behavior has been mapped to promoter 4 transcript expression in brain and ovaries [1].

We recently reported that one of the *foraging* transcript classes that originates from one of its four promoters (promoter 4) is solely responsible for regulating feeding behavior differences between two fruit fly strains, rovers and sitters [1]. When allowed to forage in an arena with sucrose drops, rovers spend more time exploring the inner area of the arena and find and consume more sucrose drops, while sitters spend more time circling the edge of the arena and find and consume less sucrose drops (Fig. 2c). Sitters have higher expression of promoter 4 *foraging* transcripts than rovers, and transgenically lowering promoter 4 expression levels in sitters switches sitter *foraging* behavior into rover. Furthermore, we found that the difference in promoter 4 expression between rovers and sitters is mediated by the epigenetic regulator *G9a*, a histone methyl transferase that differentially methylates the promoter 4 region in rovers and sitters. Rovers have higher levels of repressive *G9a*-mediated methylation at promoter 4 than sitters, resulting in lower promoter 4 expression levels in rovers than sitters.

**Figure 2.**
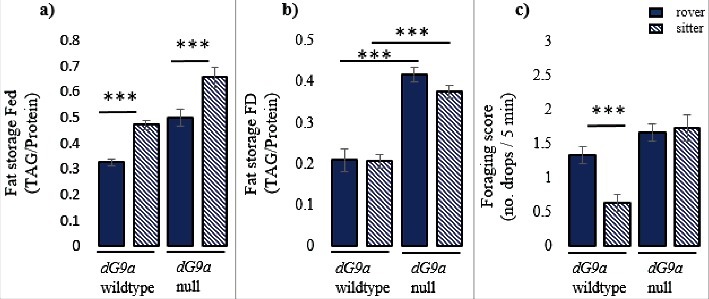
Fat stores and feeding behavior are regulated by independent mechanisms. a) Total triglyceride measurements for fed adult *D. melanogaster* females. Fed flies with the sitter *foraging* allele are fatter than flies with the rover allele, suggesting that *foraging* affects fat storage in adult flies, as it does in larvae [3]. Nevertheless, the difference in fat stores is not mediated by *G9a*, as it is maintained in the *G9a* mutant. b) – c) Total triglyceride measurements and foraging scores for 24 hr food-deprived (FD) adult *D. melanogaster* females. 24 hr FD sitters do not differ from rovers in fat stores but have significantly higher foraging scores. Although *G9a* affects fat storage in FD flies, this is not related to rover-sitter differences in feeding behavior, suggesting that fat storage and feeding behavior are independently regulated. Triglycerides (TAG) were quantified as described in [3] and standardized over total protein levels, quantified using the Pierce BCA protein assay (Thermo Scientific 23225). N = 10 with 10 flies per replicate. Statistical analysis: differences between strains were tested using one-way ANOVAs in SigmaPlot. Significance levels: *** = *p* < 0.001; ** = *p* < 0.01; * = *p* < 0.05. Error bars represents standard error of the mean (SEM). Fig. 2c is taken from [1].

Interestingly, although several of *foraging*'s other promoters show expression and methylation differences between rovers and sitters, only promoter 4 shows the differences linked to rover and sitter adult feeding behavior patterns. This suggests that the different transcripts of the *foraging* gene have distinct functions, and *foraging*'s promoter 1–3 are likely responsible for regulating other phenotypes. For instance, *foraging* also affects adult fat content in well fed flies, with flies carrying the sitter allele being fatter than flies carrying the rover allele (Fig. 2a). Nevertheless, 24 hr food-deprived rovers and sitters show no fat content differences but maintain feeding behavior differences (Fig. 2b–c), suggesting that fat content and feeding behavior are regulated independently. The rover-sitter differences in fat content are also not regulated by the *G9a*-mediated methylation and expression levels of promoter 4, since loss of *G9a* has no effect on the rover-sitter difference in fat levels (Fig. 2a–b). Furthermore, in larvae, fat content, foraging pathlength and food intake seem to be independently influenced by the *foraging* gene [3].

*foraging's* role in regulating adult feeding behavior is both transcript and tissue specific. The *G9a*-mediated expression and methylation differences at promoter 4 are found in the brain and the ovaries (Fig. 1) [1], tissues with known roles in feeding behavior in D. melanogaster [7,8]. Promoter 4 transcripts can also be found in other tissues, but without the characteristic expression differences that underlie adult feeding patterns in rovers and sitters. This difference in promoter 4 regulation across tissues is likely due to differences in co-expressed transcription factors across tissues. Furthermore, early 3^rd^ instar larvae do not show some of the promoter-specific expression differences seen in rover and sitter adult flies [3], indicating that the transcription factors that bind to *foraging*'s promoters differ not only across tissues, but also across developmental stages.

In the case of *foraging* promoter 4 regulation, the *G9a*-mediated promoter methylation and expression differences between rovers and sitters correlate with a single nucleotide polymorphism (SNP) in the promoter 4 region. From a pleiotropy perspective, the interaction between genetic variation, transcription factors, and epigenetic regulators is one of the ways in which genetically distinct individuals can display differences in some, but not all, behaviors regulated by a gene. While phenotypes associated with *foraging* promoter 4 products are expected to be influenced by SNPs in promoter 4, other *foraging-*related phenotypes will not.

More evidence supporting the hypothesis that the four promoters of *foraging* are regulated by distinct transcriptional regulators comes from comparing the promoter sequences of this gene in the rover and sitter strains. Comparing rovers and sitters, as well as the reference genome line for *D. melanogaster*, the highest sequence variation in this gene lies in the promoter regions. In the case of promoter 4, which only has one polymorphism between rovers and sitters, this polymorphism falls on a putative binding site for the transcription factor *mad* [1]. Furthermore, the 4 promoters differ in transcription factor binding site type and number (Table 1). Likely it is the specific combination of different transcription factor binding sites with the cellular environment (i.e. what transcription factors are being expressed in a specific tissue at a specific time) that drives the tissue and time-specific expression of the *foraging* promoters.

**Table 1.** Putative transcription factor binding sites in *foraging* promoter (Pr1-4) regions 500 bp upstream of transcription start site, predicted by PROMO [11] within a dissimilarity margin less than 1%.

	Transcription factor												
	*Ftz*	*T11*	*Mad*	*Hb*	*Prd*	*DSXM*	*DSXF*	*Eve*	*Zen1*	*Zeste*	*Dfd*	*B factor*	*GAGA factor*
Pr1	14	6	—	3	2	1	1	—	—	—	—	—	—
Pr 2	3	1	1	1	—	—	—	1	1	1	—	—	—
Pr 3	3	—	4	—	1	—	—	—	—	—	—	—	—
Pr 4	6	2	2	1	1	—	—	—	—	—	1	1	1

## Conclusions

In sum, there are multiple levels at which molecularly complex genes can achieve pleiotropic effects. First, multiple transcripts with distinct open reading frames (i.e. distinct protein products) can have distinct downstream targets. For example, although *foraging*'s 21 transcripts all encode the same kinase domain, they vary substantially in their substrate binding and dimerization domains. Second, independently regulated promoters can drive expression of different gene products in different tissues at different times during development, or in response to different environmental stimuli. Independent expression of patterns of promoters is likely achieved by different transcription factor binding sites in the promoter sequences. Third, the cellular environment influencing where and when a gene product is expressed (i.e. in what tissue, at what time) will determine if and how it is post-transcriptionally regulated. For instance, there is some data to suggest that the RNA-binding protein *pumilio* may post-transcriptionally regulate *foraging* but can only do so in tissues where it is co-expressed [9,10].

Deciphering the mechanism underlying the regulation of genes with multiple effects is important because secondary effects of a gene can confound results when studying a phenotype. Multiple independent phenotypic effects and a decidedly complex molecular structure make the *foraging* gene an excellent model for the study of pleiotropy.
